# Towards Prediction of Metabolic Products of Polyketide Synthases: An *In Silico* Analysis

**DOI:** 10.1371/journal.pcbi.1000351

**Published:** 2009-04-10

**Authors:** Gitanjali Yadav, Rajesh S. Gokhale, Debasisa Mohanty

**Affiliations:** National Institute of Immunology, New Delhi, India; UT Southwestern Medical Center, United States of America

## Abstract

Sequence data arising from an increasing number of partial and complete genome projects is revealing the presence of the polyketide synthase (PKS) family of genes not only in microbes and fungi but also in plants and other eukaryotes. PKSs are huge multifunctional megasynthases that use a variety of biosynthetic paradigms to generate enormously diverse arrays of polyketide products that posses several pharmaceutically important properties. The remarkable conservation of these gene clusters across organisms offers abundant scope for obtaining novel insights into PKS biosynthetic code by computational analysis. We have carried out a comprehensive *in silico* analysis of modular and iterative gene clusters to test whether chemical structures of the secondary metabolites can be predicted from PKS protein sequences. Here, we report the success of our method and demonstrate the feasibility of deciphering the putative metabolic products of uncharacterized PKS clusters found in newly sequenced genomes. Profile Hidden Markov Model analysis has revealed distinct sequence features that can distinguish modular PKS proteins from their iterative counterparts. For iterative PKS proteins, structural models of iterative ketosynthase (KS) domains have revealed novel correlations between the size of the polyketide products and volume of the active site pocket. Furthermore, we have identified key residues in the substrate binding pocket that control the number of chain extensions in iterative PKSs. For modular PKS proteins, we describe for the first time an automated method based on crucial intermolecular contacts that can distinguish the correct biosynthetic order of substrate channeling from a large number of non-cognate combinatorial possibilities. Taken together, our *in silico* analysis provides valuable clues for formulating rules for predicting polyketide products of iterative as well as modular PKS clusters. These results have promising potential for discovery of novel natural products by genome mining and rational design of novel natural products.

## Author Summary

Polyketide synthases (PKSs) form a large family of multifunctional proteins involved in the biosynthesis of diverse classes of therapeutically important natural products. These enzymes biosynthesize natural products with enormous diversity in chemical structures by combinatorial use of a limited number of catalytic domains. Therefore, deciphering the rules for relating the amino acid sequence of these domains to the chemical structure of the polyketide product remains a major challenge. We have carried out bioinformatics analysis of a large number of PKS clusters with known metabolic products to correlate the chemical structures of these metabolites to the sequence and structural features of the PKS proteins. The remarkable conservation observed in the PKS sequences across organisms, combined with unique structural features in their active sites and contact surfaces, allowed us to formulate a comprehensive set of predictive rules for deciphering metabolic products of uncharacterized PKS clusters. Our work thus represents a major milestone in natural product research, demonstrating the feasibility of discovering novel metabolites by *in silico* genome mining. These results also have interesting implications for rational design of novel natural products using a biosynthetic engineering approach.

## Introduction

It is well known that polyketide synthase (PKS) gene clusters can generate enormously diverse array of polyketide products by making use of various biosynthetic paradigms like, modular organization of sets of catalytic domains or iterative catalysis of condensation steps using single set of catalytic domains [1]. In view of the pharmaceutical importance of polyketides, there is tremendous interest in identifying PKS gene clusters capable of producing novel polyketides by genome mining. However, the relating the sequence of the various catalytic domains present in a PKS biosynthetic cluster to the chemical structure of the final metabolic product is a major challenge. The availability of the sequences of a large number of experimentally characterized PKS clusters and 3D structural information on homologous protein domains presents a unique opportunity to carry out *in silico* analysis for addressing structural and mechanistic issues concerning polyketide biosynthesis. A number of recent theoretical studies have demonstrated the utility of *in silico* analysis in providing novel insights into the mechanistic details of polyketide biosynthesis as well as in identifying novel natural products by genome mining. Computational analysis of polyketide synthase (PKS) and nonribosomal peptide synthetase (NRPS) proteins have provided valuable clues for development of knowledge-based methods for identification of catalytic domains in PKS [2],[3] and NRPS [4] proteins, prediction of the substrate specificity for AT domains [2],[3],[5] and adenylation domains [4],[6],[7]. Such predictions have also been experimentally validated by the recent successful reprogramming of the phthiocerol dimycocerosate (PDIM) biosynthetic pathway in *Mycobacterium tuberculosis* [8] and experimental characterization of a novel exogenous standalone enoyl reductase (ER) involved in PDIM biosynthesis [9]. Bioinformatics analysis of secondary metabolite biosynthetic pathways have also played a crucial role in discovery of novel natural products by genome mining [10]–[14]. Very recently it has also been demonstrated that, computational analysis of KS domains from trans-AT PKS clusters can give novel clues about the chemical structures of the final polyketide product [15]. Similarly, bioinformatics analysis of docking domain sequences (the original term applied to these regions was “interpolypeptide linker”, but the term docking domain is being increasingly used in recent literature) have given novel insight into the evolution of specificity in inter polypeptide interactions in modular PKSs [16]. Pioneering work at Ecopia BioScience using data mining approaches has also led to development of proprietary databases which can aid in genomics driven discovery of cryptic biosynthetic pathways [17] and utility of these databases have been demonstrated by identification of novel secondary metabolites [18].

Thus, these studies have established that knowledge based computational approaches can play a powerful role in elucidation of novel secondary metabolite biosynthetic pathways. However, for *in silico* identification of polyketide products of uncharacterized PKS clusters, the computational method should also take into consideration various different paradigms employed by PKS biosynthetic machinery [19]. Several excellent reviews [20],[21] describe the type I, type II and type III biosynthetic paradigms. Type I modular PKSs harbor distinct sets of catalytic domains, each set termed as a “module”. Each module is responsible for one condensation step and the number of modules in a modular PKS correlate directly with the number of ketide units in its biosynthetic product. In contrast, type I iterative PKSs are characterized by a single set of catalytic active sites which are used iteratively for several rounds of successive condensations till the final product is released. It was initially believed that bacterial PKSs are modular while fungal PKSs function in an iterative manner. However, discovery of mixed PKS clusters involving programmed iterative modules and several other deviations [22],[23] from conventional textbook PKS biosynthetic paradigms in various microbes indicate that PKS proteins are not amenable to simple classification based on species of their origin. Therefore, *in silico* methods should be capable of predicting from sequence information, whether a given PKS cluster is iterative, the number of iterative chain condensation steps catalyzed by it and crucial amino acids which control the number of iterations.

In contrast to type I iterative PKSs where a single multifunctional enzyme is involved in biosynthesis of the polyketide product, biosynthesis in type I modular PKS clusters often involve multiple ORFs, each containing several modules. Therefore, predicting the correct order of substrate channeling between various ORFs is crucial for deciphering the final metabolic product of a modular PKS cluster. Several lines of experimental evidence reveal that inter subunit interactions between C-terminal docking domain region of the upstream ORF and N-terminal docking domain region of the downstream ORF, play a crucial role in channeling of substrates from upstream domains to downstream domains [24]–[27]. Moreover, these interactions involving C-terminus and N-terminus amino acid stretches have been reported to increase the maximum velocity (k_cat_) of chain transfer of otherwise disfavored substrates by as much as 100-fold [28]. Structural studies using NMR suggest that, these terminal docking domain regions of PKS proteins adopt a specific 3-dimensional fold consisting of a four helix bundle structure [29]. In fact, after the elucidation of this NMR structure, the term ‘docking domain’ is being increasingly used in the recent literature to describe these terminal amino acid stretches, which were earlier called ‘inter polypeptide linkers’. Based on this structure, it has been proposed that recognition between upstream and downstream ORFs in a modular cluster is governed by formation of specific contacts in the docking domain. Several recent experimental studies [30],[31] have further validated the role of specific inter polypeptide contacts in controlling inter subunit communication in modular PKS clusters. Very recently NMR studies [32] have also elucidated the role of similar docking domains in governing protein-protein interactions in hybrid megasynthases. Even though these experimental studies have identified specific residue pairs involved in inter subunit recognition, no systematic analysis of experimentally characterized modular PKS clusters have been characterized to investigate whether correct order of substrate channeling in type I modular PKS clusters can be predicted based on these specific inter polypeptide contacts. It may be noted that, even though recent study by Thattai *et al* [16] has attempted to address this question, their algorithm for prediction of PKS multiprotein chain order has been tested on a hypothetical five ORF cluster with only six combinatorial possibilities.

In this work, we have carried out a detailed comparative analysis of the experimentally characterized modular and iterative PKS clusters with known polyketide products to address following major questions relating to *in silico* prediction of polyketide products. Is it possible to distinguish between modular and iterative PKS from their sequence alone? Can we predict the number of iterations a given iterative PKS protein would catalyze and identify crucial amino acid residues that control the number of iterations? Is it possible to predict the correct order of substrate channeling between various ORFs in a modular PKS cluster? We have carried out profile Hidden Markov Model (HMM) analysis of KS domains to identify signature profiles which can decipher whether a PKS protein is modular or iterative. Structural modeling of KS domains of iterative PKS proteins and analysis of their active site pockets have given novel insight into the structural features that dictate the number of iterations catalyzed by a PKS protein and crucial amino acids which control them. Similarly, comparative analysis of crucial inter polypeptide contacts between cognate and non-cognate pairs of ORFs based on the three dimensional structure of the docking domains have given novel clues for prediction of the correct order of substrate channeling.

## Results

### Distinguishing between modular and iterative PKSs

KS domains are the most conserved among various catalytic PKS domains and are responsible of catalysis of the chain condensation step. We have analyzed them in detail to identify class specific conserved patterns which distinguish modular and iterative PKS systems. For KS domains, the total dataset comprised of 217 pure modular KS domains, 82 pure iterative domains, 19 enediyne, 43 trans-type and 34 KS domains from hybrid NRPS-PKS clusters. Apart from the sequences of 20 experimentally characterized bacterial type I modular clusters included in our earlier analysis [2], an additional set of 18 modular PKS clusters was used as described in Methods. Despite sharing a significant degree of homology ranging from 24% to 40% sequence identity, KS domain counterparts from modular and iterative PKSs and other PKS subfamilies, segregate into distinct clusters in a phylogenetic dendrogram (Figure S1). We have used profile Hidden Markov Models (HMMs) to quantify subtle position specific differences in the probability of occurrence of amino acids in various subfamilies of KS domains (See **methods** for description of various subfamilies). The available KS data set was divided into training and test set, and sequences belonging to the training set were used for building profile Hidden Markov Models by the HMMER package [33]. Benchmarking on the test set indicated that, these HMM profiles were highly sensitive, with a prediction accuracy of 100% for both enediyne and trans-AT sub families, 97% for pure iterative PKSs, 92% for modular KS domains and 88% for hybrid clusters. Therefore, using HMM profiles it is not only possible to distinguish between modular and iterative PKS with a very high accuracy, these profiles can also be used to classify an uncharacterized sequence of a KS domain into various subfamilies within modular and iterative systems. This result has interesting implications for genome sequencing efforts towards identification of novel PKS clusters, because from KS sequence alone, one can get clues about PKS family and decide whether to sequence the entire cluster or not.

### Identification of sequence and structural features that control number of iterations

**Figure 1:**
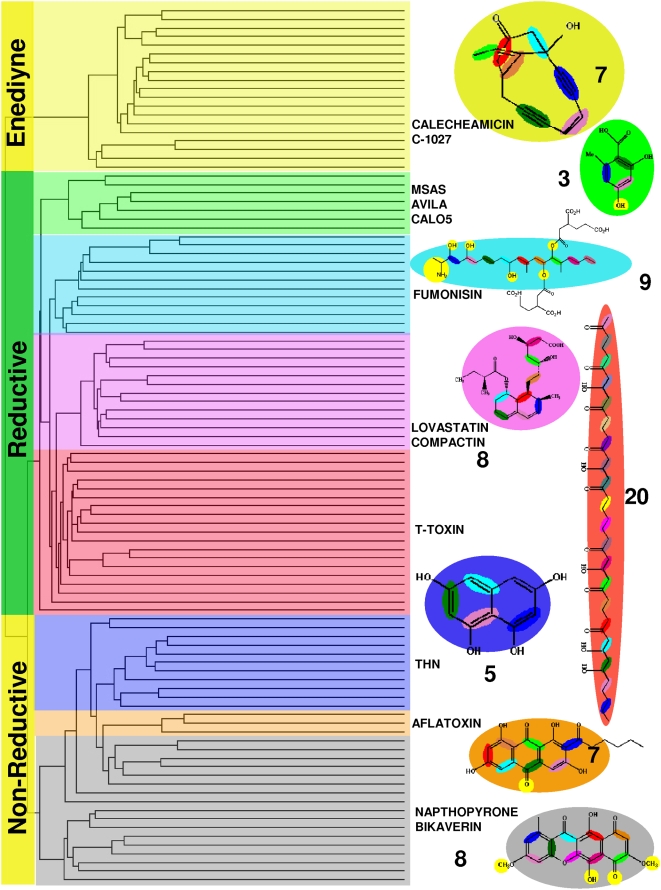
Dendrogram of KS domains from type-I iterative PKS clusters. The branches of the dendrogram have been colored according to the number of iterations catalyzed by the corresponding KS domain. The corresponding polyketide structures have been depicted in the same color.

**Figure 2:**
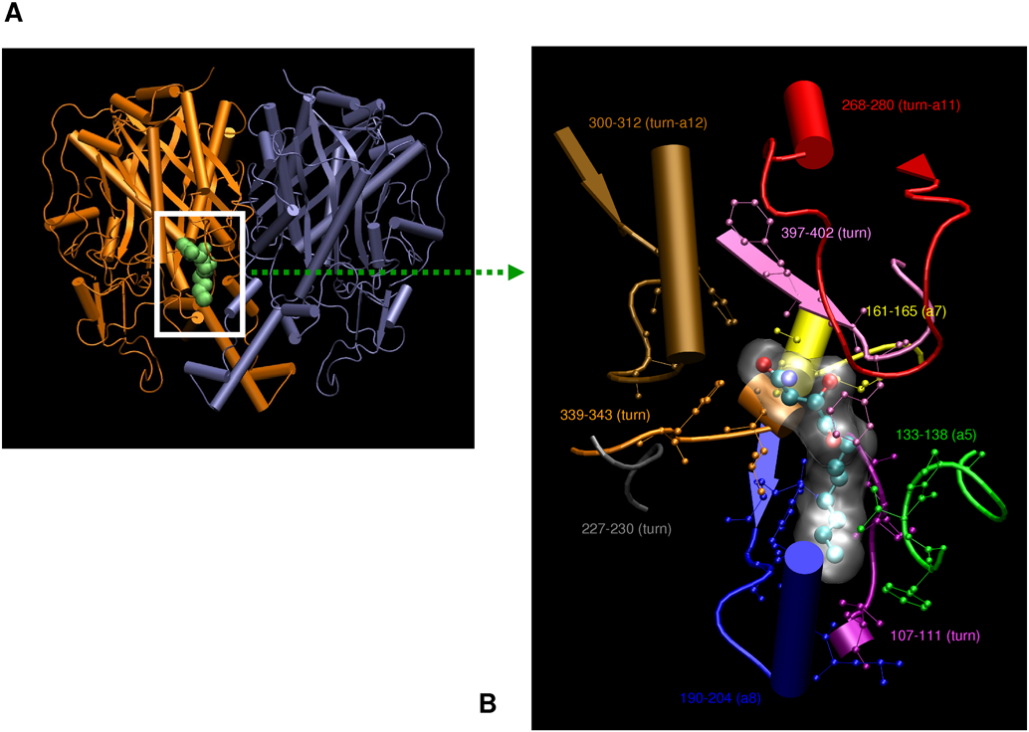
Structural template for modeling of iterative KS domains. (A) The *E. coli* KAS-II homo-dimer with ligand. (B) The backbones (secondary structural rendering) and side chains (ball and stick) of different stretches of amino acids that constitute the ligand binding cavity of *E.coli* KAS-II have been depicted in different colors.

The polyketide products of various iterative PKS proteins are biosynthesized by different number of iterative condensation steps and undergo varying degrees of reductions. Phylogenetic analyses of iterative KS domains revealed that the clustering of iterative PKS sequences is highly correlated with the number of iterations they perform and degree of reductions undergone by the metabolite during biosynthesis (Figure 1). The biosynthesis of polyketides, lovastatin and bikaverin involve eight condensation steps, but their final structures are different because of the different cyclization patterns. Our analysis suggests that, the sequence of KS domain encodes information about chemical structure of the polyketide product. Hence, KS sequences of lovastatin and bikaverin form two different clusters. Based on similar phylogenetic analysis, earlier reports have proposed that KS domains cluster into groups depending on whether the corresponding type I iterative PKS contains additional reductive domains [34]–[36]. We attribute this feature to a complex programming within the KS domains which enables specific molecular recognition of the products. The observed clustering in Figure 1 could thus be arising from sequence features, that control recognition of specific substrates which have undergone different degrees of chemical and structural modifications due to the presence of reductive domains. Therefore, we wanted to analyze the structural models of various iterative KS domains for identification of specific amino acids or sequence stretches that can potentially control substrate size and extent of unsaturation. The various iterative KS domains were modeled using comparative modeling approach (see Methods for details). The structural templates for various iterative KS domains were identified by BLAST search against PDB or by using threading approach. The *E. coli* KAS-II protein (pdbids 1KAS, 1B3N) were used as the templates for modeling these iterative KS domains. Since 1B3N was a ligand bound structure (Figure 2A), the putative active site pockets (Figure 2B) of various iterative KS structural models could be identified based on amino acids which were in contact with the bound ligand in 1B3N. The structural features of the active site pockets of different iterative KS domains were analyzed further to identify the cavity lining residues (CLRs) and cavity volumes following protocols described in the methods section. Active site residue patterns (Figure 2B) in these structural models allowed us to correlate the cavity volume and hydrophobicity of the active site pockets to the number of iterations and the degree of unsaturation of the polyketide products they synthesize.

**Figure 3:**
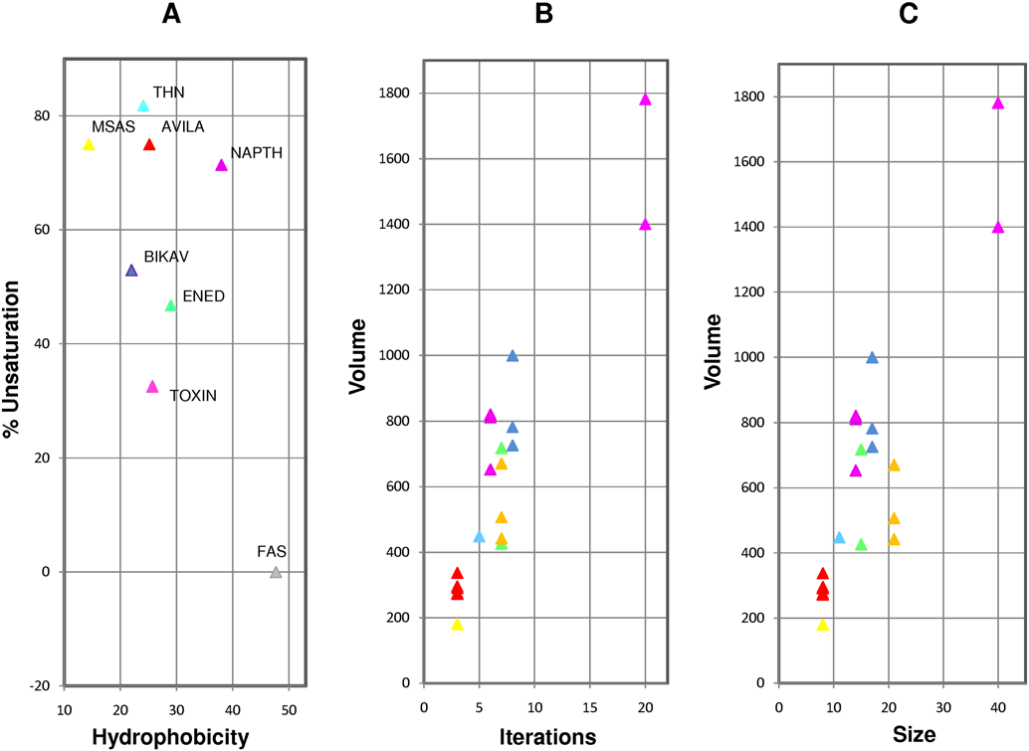
Variation in hydrophobicity and size of the active site cavities of various iterative KS domains. The KS domains carrying out different number of iterations have been depicted in separate colors. Points corresponding to different homology models of the same KS domain have a common color. Hydrophobicity of CLRs correlates negatively with the extent of unsaturation in the final product (A). Cavity volumes (Å^3^) correlate positively with the number of iterations (B). Cavity volumes (Å^3^) of iterative KS domain pockets show a positive correlation with final product size (number of backbone carbon atoms in the polyketide) (C).

**Figure 4:**
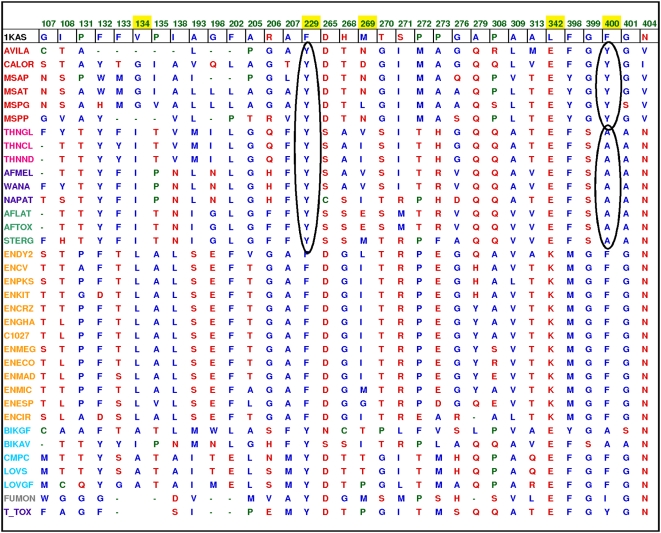
List of residues lining the active site pockets of KS domains in various iterative PKS clusters. For clarity, positions that have completely invariant residues (for e.g. the catalytic triad) or positions with a high number of gaps have been removed from this table. The highlighted positions have been discussed in detail in the text, and are likely to govern the carbon chain length in different iterative PKSs. The two crucial positions, 229 and 400 have been circled.

**Figure 5:**
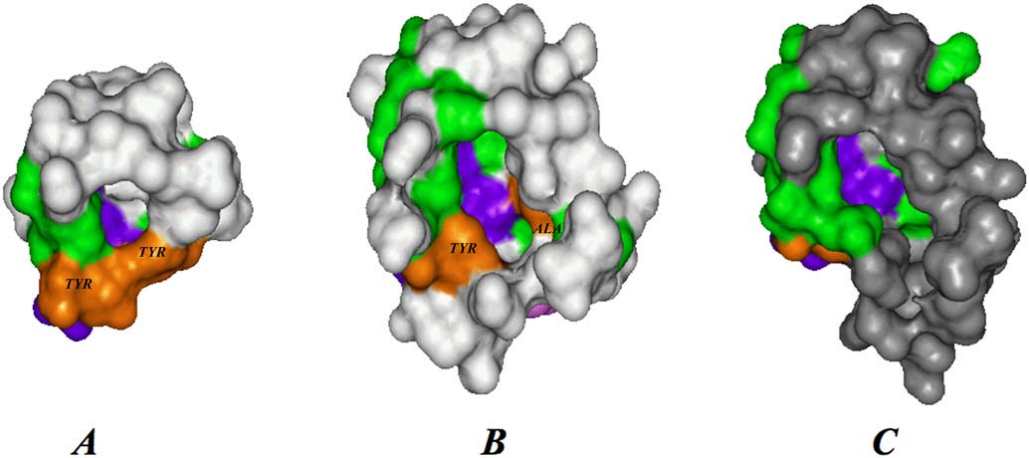
Functionally important cavity lining residues of two types of iterative KS domains. MSAS (A) and NAP (B). The cavities of the models have been shown in surface rendering. Each model has been superimposed with the structural template. The two orange residues correspond to the positions 229 and 400, which together block the downward flow of the MSAS cavity. One of these residues is an Ala in case of the intermediate NAP-type cavity and this allows the cavity to flow downwards. These cavities are actually buried inside the protein, and residues forming the top layer have been removed for clarity. (C) The internal topology of the structural template, *E. coli* KAS-II protein cavity has been depicted for reference. The surface has been colored such that the catalytic triad is in purple, regions which are invariant among the different iterative KS domains, are in green. Thus the differences in the cavity shapes arise from residues lying in the grey region of the depicted cavity surface. The cavity is completely buried, but the top layer of residues has been removed for clarity of the figure.

The substrate binding cavity in the 1KAS is highly hydrophobic owing to its completely saturated substrate. Polyketides, on the other hand, may contain several hydroxyl groups and unsaturated double bonds. Accordingly, the catalytic pockets in the structural models of polyketide KS domains were found to be less hydrophobic compared to the FAS cavities. Table 1 compares PKS product characteristics with a variety of cavity features. We observed a distinct difference in pocket hydrophobicity within polyketides and it correlated negatively with the extent of unsaturation seen in the product (Figure 3A). For example, the T-toxin PKS model cavity is more hydrophobic than the methylsalicylic acid synthase (MSAS) model cavity and this correlates with the fact that T-toxin is a reducing PKS having a greater proportion of saturated carbons in its final product than the partially reducing MSAS polyketide. Interestingly, cavity volumes correlate positively with the number of iterations (or corresponding product size). We found that polyketide KS cavity volumes fall into three distinct groups; small, large and intermediate (Figure 3B and 3C). The smallest cavities (∼300Å^3^) belong to the MSAS type PKSs that perform three iterations. Intermediate sized cavities (∼800Å^3^) belong to the napthopyrone (NAP) like PKSs that iterate from five to eight times. The largest cavities, 1780Å^3^, were observed for the T-Toxin models that perform 20 iterations. Figure 2B depicts the residues that line the hydrophobic cavity of the template KAS-II protein (volume 934 Å^3^) and surround the ligand analogue cerulenin. A comparison of the modeled structures with the template FAS KS structure revealed that in case of MSAS and NAP, the backbones of the models had not altered significantly during modeling (Figure S2), and thus, their functional difference could be traced to specific cavity lining residues (CLRs) (Figure 4). Figure 5A and 5B show the surface topology of the small and intermediate sized cavities. Figure 5A depicts the modeled MSAS KS domain with two tyrosines protruding into the KS cavity from opposite walls and thus blocking the downward flow of the cavity along the dimer interface. These two cavity blocking residues correspond to positions 229 and 400 (1KAS numbering). Interestingly, the conservation profiles of the CLRs shown in Figure 4 revealed that these two *Tyr* residues are highly conserved in all PKSs which carry out three iterations. This further substantiates the important role attributed to these residues based on our structural modeling of the active site pocket. Remarkably, NAP type KS domains have an *Ala* at position 400, that allows the cavity to extend further down thus making their cavities similar to the FAS catalytic cavity, shown for reference in Figure 5C.

**Table 1:** Comparison of the cavity volumes and hydrophobicities of various KS structural models with the number of iterations and product size.

		Product Size (No. of backbone carbons )	Number of iterations	Cavity Volume (Å^3^)	Number of hydrophobic residues	Hydrophobicity	Number of CLRs
FAS	Reducing	Variable	Variable	934	18	47.7	47
MSAS	Partial	8	3	180	9	14.4	24
AVILA	Partial	8	3	291	8	25.2	20
THN	Non-reducing	10	5	819	12	24.1	32
WA-NAP	Non-reducing	14	6	895	16	38	44
T-TOXIN	Reducing	40	20	1781	16	25.7	56

CLR: Cavity Lining Residues.

Structural analysis thus revealed how substrate binding sites of varying size and hydrophobicity can be generated in type I iterative KS domains by subtle variations of residues on similar backbone folds. The crystal structure of KS-CLF also highlights how specific residues can regulate chain length in type-II PKSs [37]. Our results on role of cavity volume in controlling number of iterative condensations or chain length of type I iterative PKS products is also supported by recent experimental studies involving swapping of KS domains in fungal iterative PKSs, where replacement of fumonisin KS domain by KS from lovastatin LDKS resulted in polyketides having short chain length [38]. Very recent experiments involving generation of altered fatty acid-polyketide hybrid products by rational manipulation of benastatin biosynthetic pathway [39] also suggest that number of chain elongations is dependent on the size of the PKS enzyme cavity. The *in silico* analysis of the sequence and structural features of iterative KS domains reported here provides a structural rationale for these experimentally observed variations in substrate specificities and further helps in identification of residues that can be specifically mutated to control the number of iterations in type-I PKSs. No experimental studies have as yet been reported on altering the number of iterations in type-I PKSs by site directed mutagenesis. The present *in silico* analysis gives crucial leads for such experiments.

### Predicting the order of substrate channeling in modular PKS clusters

In modular PKS clusters, the chemical structure of the product is governed by the order in which substrates are channeled between various ORFs. It has often been observed that the order of PKS ORFs during biosynthesis of a polyketide is not the same as the order of the corresponding ORFs in the genome. This complexity of module succession has been depicted in Figure S3 using schematic representation of a type I modular PKS cluster. This biosynthetic cluster has four polyketide synthase ORFs and their order in the genome is Orf1, Orf2, Orf3 and Orf4. But during the biosynthesis, Orf4 is the first to function and the product of Orf4 is transferred to Orf1. Orf2 functions at a later stage and its product is condensed with the rest of the polyketide. This inconsistency between ordering of ORFs in the genome and the order of substrate channeling is a commonly observed phenomenon, as is evident from the simocyclinone [40], nanchangmycin [41], microcystin [42], pimaricin, rapamycin and nystatin biosynthetic clusters. The prediction of the correct order of substrate channeling is essential for *in silico* identification of polyketide products of uncharacterized modular PKS clusters. Therefore, deciphering the cognate combination of ORFs in a modular PKS cluster from the large number of theoretically possible non-cognate combinations has been the major bottleneck in formulating predictive rules for *in silico* identification of polyketide products. Hence, we attempted to investigate whether predictive rules based on specificity of interaction between ORFs can be formulated for deciphering the correct order of substrate channeling in an uncharacterized PKS cluster.

Several experimental studies have suggested that inter protein interactions in modular PKSs are mediated by specific recognition between docking domains or the so called ‘interpolypeptide linker’ regions [24],[25],[29]. The amino acid stretches N-terminus to the first KS domain and C-terminus to the last ACP domain are referred as inter polypeptide linkers or docking domains. These have been extensively studied and it has been proposed that, the C-terminal (Cter) docking domains specifically pair with the N-terminal (Nter) docking domains of the succeeding ORF to facilitate cross-talk between the consecutive ORFs. Structural elucidation [29] of the cognate docking domains from erythromycin PKS (DEBS) has revealed that, unlike conventional linker sequences which join protein domains covalently within polypeptides, these docking domain regions are not non-structured, but adopt a relatively compact four helix bundle structure. It has been proposed that, this four helix bundle structure is the core fold of cross-talk [29] between ORFs of modular PKS clusters. These structures have been termed inter protein ‘docking domains’ to emphasize that they are responsible for the recognition and subsequent docking between successive protein modules. The C-terminal docking domain is reported to contain three helices (hereafter named helix 1, 2 and 3) whereas the N-terminal docking domain contains a single longer helix (hereafter named helix 4). This docking domain complex is a symmetrical dimer, consisting of two independent structural units called domain A and domain B. Domain A is an unusual intertwined α-helical bundle comprising helices 1 and 2. Domain B is also an α-helical bundle but with an entirely different topology and it comprises helix 3 (from Cter) and helix 4 (from Nter). Thus the actual docking interaction occurs in domain B, via several pairs of charged residues and a conserved set of hydrophobic residues. However, it has been proposed that, out of these various interacting residues, two pairs of appropriately placed charged residues at critical positions on the docking interface, form a kind of ‘docking code’ for DEBS [29] (Figure S4). When DEBS1 docks against DEBS2, the charges at these positions give rise to favorable interactions. However, in case of non-cognate combinations between DEBS1 and DEBS3, the resulting charge interactions are repulsive. The availability of DEBS docking domain structure provided us the opportunity to test, whether such a code exists in other PKS systems as well. We have carried out a structure based analysis of docking domain sequences to investigate if rules for identification of cognate ORF combination can be formulated based on key interactions found in DEBS docking domain structure.

**Figure 6:**
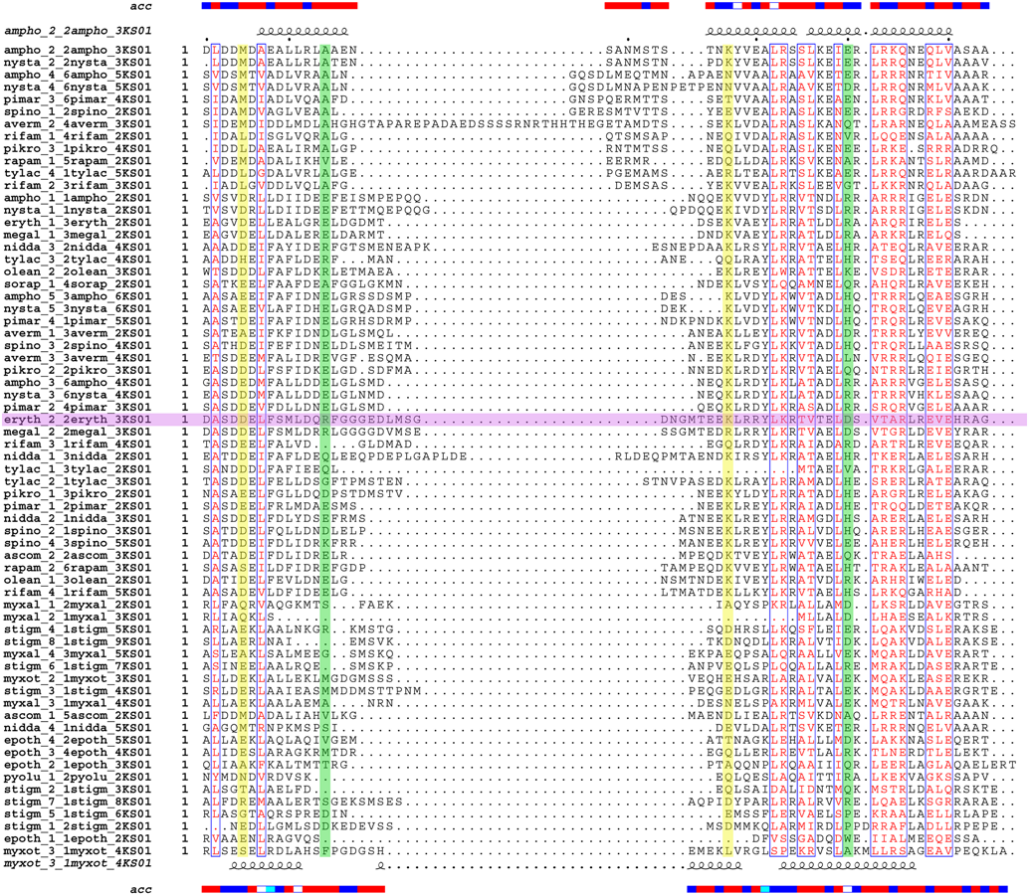
A structure based sequence alignment of the docking domains from various PKS clusters. Helix 3 and helix 4 were concatenated before secondary structure prediction. ESPript service [89] from the predict protein server was used for structural based sequence alignment of docking domains. The N-terminus docking domain consists of the sequence stretch extending from N-termini to the beginning of the first KS domain, while the C-terminus docking domain extends from the end of the last ACP domain to the C-terminus of the PKS protein. Inter polypeptide contacts were extracted using the DEBS NMR structure as a template. The two pairs of interacting residues which constitute the docking code have been highlighted in green and yellow respectively. The reference sequence of DEBS docking domains is highlighted in purple color.

It may be noted that, based on bioinformatics analysis of docking domains in type I modular PKS proteins, Broadhurst *et al* [29] had also proposed that DEBS-like docking domain structures would be present in other type I modular PKS clusters and they govern the cross-talk between ORFs. Since secondary structure analysis by Broadhurst *et al* [29] had clearly demonstrated propensity of docking domain sequences for four helix bundle structure similar to DEBS docking domain, inter polypeptide contacts were extracted for both cognate and non-cognate pairs of ORFs in various modular PKSs using the DEBS docking domain structure as a template. Since recent studies [16],[29],[43] suggest that PKS docking domains fall into at least three different phylogenetic classes, our assumption regarding docking domains from various phylogenetic groups adopting similar structural folds requires further justifications. It is well known that for a given protein family, structure is conserved to a much larger extent than sequence [44],[45]. There are many examples of proteins adopting similar three dimensional structural fold even in absence of detectable sequence similarity [44],[45]. Recently available structures [46] of mammalian type I FAS proteins also show remarkably high similarity to structures PKS protein domains even if they share only a limited sequence homology. Therefore, our assumption regarding myxobacterial PKS ‘docking domains’ adopting structural folds similar to docking domains from actinomycetes is not unreasonable. Hence, we extracted crucial interacting residues for various docking domain pairs based on alignment with DEBS docking domain structure. Figure 6 shows the alignment of cognate pairs of various PKS docking domain sequences with DEBS docking domain structure. The interacting residue pairs obtained from this alignment were ranked as favorable, unfavorable or neutral as per a simple scoring scheme (Table S1). The interactions between a pair of oppositely charged amino acids or between a pair of hydrophobic amino acids were ranked as favourable, while electrostatic repulsions between a pair of charged amino acids was called unfavourable. On the other hand, interactions between any other amino acid pairs, specifically the interactions between charged and hydrophobic amino acids was ranked as neutral. It may be noted that, this simplistic scoring scheme has been defined based on types of amino acid contacts found in interfaces of protein-protein complexes [47]. A total of 66 cognate pairs of docking domain sequences were checked for the two pairs of positions which give rise to favorable electrostatic interactions in the docking domain structure. Out of these, 54 pairs of ORFs were found to have at least one residue pair with favorable interaction. Moreover, there was no cognate pair where both of these interactions were unfavorable. Thus it can be concluded that cognate pairing of ORFs does generate energetically favorable contacts.

**Figure 7:**
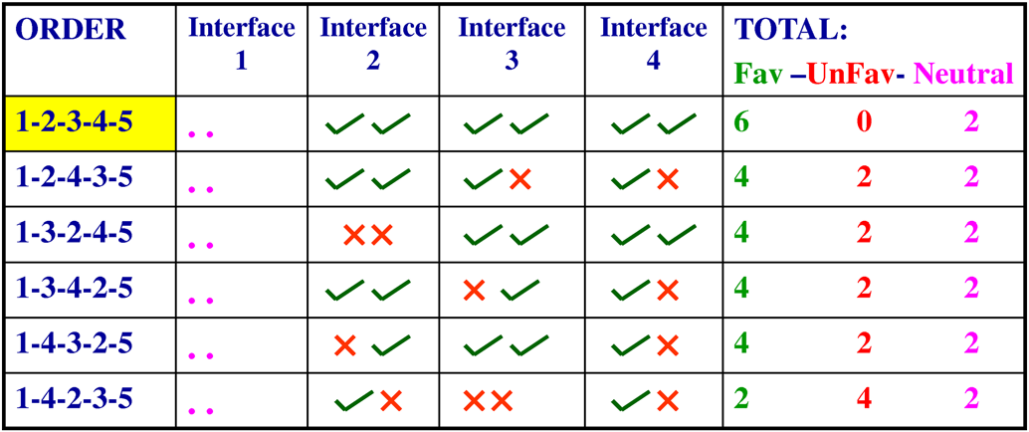
List of various combinatorial possibilities for the order of substrate channeling in the Spinosad modular PKS cluster. The Spinosad PKS has five ORFs which can be arranged in six different combinations, if the identity of the first and last ORF is fixed. This has been shown in the first column, where the native or correct order of ORFs has been highlighted. Each combination has four possible interfaces and each interface has been scored for two pairs of critical contacts. These two interactions can be favorable (green tick mark) or unfavorable (red cross mark) or neutral (pink dot). The last column shows the total number and type of contacts. The combination of ORFs with the highest number of favorable contacts and lowest number of unfavorable contacts is assigned as the best scorer. As can be seen, the native combination is the highest scorer in this case.

Since a good docking code interaction was observed in more than 80% cases, we investigated if these crucial inter polypeptide contact pairs could be used to predict the correct order of module succession in a given modular PKS. If all possible combinations of ORFs in a PKS cluster are considered together, there would be only one biosynthetically correct order of ORFs. This correct combination would in turn have a set of all cognate interfaces and therefore, the highest number of favorable interactions. The remaining combinations of ORFs would be incorrect and accordingly, they would have varying numbers of non-cognate interfaces, thus resulting in unfavorable interactions. It may be added here that, the identity of the first and last ORFs can usually be established by the presence of an initiating loading module and the terminal TE domain respectively. The presence of a very short C-terminal sequence beyond the conserved TE domain can also be used as a criterion for identification of the last module. Figure 7 shows the example of the Spinosad biosynthetic cluster which has ten modules arranged in five ORFs. These five ORFs can be combined in six different ways if the first and last ORFs are fixed. Each of the six combinations would have four interfaces. All the interfaces were scanned for favorable, unfavorable or neutral interactions at the positions corresponding to the DEBS docking code. As can be seen in Figure 7, the correct order of ORFs has the highest number of favorable interactions and no repulsive interaction at any of its interfaces. In contrast, each of the remaining five combinations has at least two repulsive interactions, and thus can be rejected in comparison with the correct combination.

**Figure 8:**
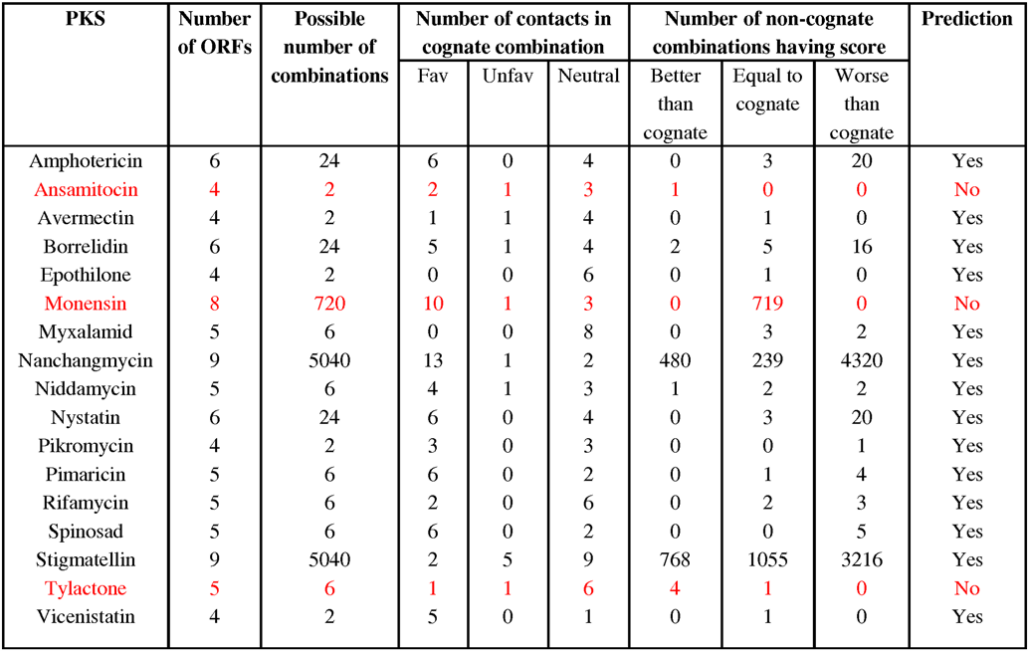
Result of the docking code analysis. The first two columns depict a PKS cluster and its corresponding number of ORFs. The third column shows the total number of ORF combinations possible, of which only one is the correct (or native) order. All possible combinations were tested for the presence of two critical interactions. The fourth and fifth columns have been further divided into three sub-columns each. The fourth column shows the interaction score (favorable, unfavorable and neutral) for the correct order of ORFs. The fifth column depicts the number of non-native combinations which resulted in a score that was better than, same or worse than native. Rows colored red depict the cases where this prediction method failed.

A total of 39 characterized PKS clusters were analyzed in this manner to test the validity of this assumption. For a representative set of PKS clusters, Figure 8 shows in tabular format, the number of favorable, unfavorable and neutral contacts in the cognate combination and also the number of non-cognate combinations having a score better, equal or worse compared to the cognate combination. As can be seen from Figure 8, in several modular PKS clusters unfavourable interactions are present. However, the number of unfavourable interactions is much smaller than the favourable or neutral interactions present in the cognate interfaces. Thus analysis of cognate inter polypeptide contacts in 17 modular PKS clusters suggest that, both the interactions need not be favourable for effective docking domain interactions. However, non-cognate interfaces have more number of unfavourable interactions. Hence, there are relatively few non-cognate combinations having a score better than cognate combination. In ten out of 17 PKS clusters, no non-cognate combination has better score than the cognate combination. Even though there are non-cognate combinations having scores equal to cognate combination, the cognate combination can still be ranked among top few in these 10 cases. In case of four other PKS clusters, there are a significant number of non-cognate combinations having score higher then the cognate combination. However, the cognate combination can still be ranked within top 20% of all possible combinations. For example, in case of nanchangmycin 480 non-cognate possibilities have better score than cognate, 239 have scores equal to the cognate combination. Thus the cognate combination is ranked in top 720 combinations. However, the total number of combinatorial possibilities is 5040. Therefore, our computational method ranks the cognate combination in top 14% in case of nanchangmycin PKS cluster. It is important to note that, despite the large number of combinatorial possibilities, prediction based on docking domain sequences alone is able to reject a sufficiently high number of non-cognate combinations. Thus, our results on analysis of docking domain sequences indicate that, in more than 80% of the cases the cognate order of substrate channeling can be predicted correctly. However, we must clarify that, ‘correct prediction’ would mean eliminating significant number of non-cognate combinations and restricting the cognate combination to a relatively smaller number of possibilities. Such a relaxed definition of ‘correct prediction’ can be justified by the fact that, we are using a simple prediction method involving few crucial contacting residues rather than all the interactions present in the docking domain structure. Secondly, we are not taking into account role of other catalytic domains in preventing chain elongation in case of non-cognate associations.

Even though very recent theoretical studies [5],[16] have attempted to predict physical interaction between PKS proteins based on analysis of co-evolution of docking domain sequences, the prediction accuracy for order of substrate channeling has either not been studied in detail [16] or found to be low in cases involving clusters consisting of more than four ORFs [5]. However, in contrast to these purely sequence based methods, we have used a structure based approach. Using the conserved core structure of the docking domain as template, we have extracted crucial interacting residues which were suggested earlier by Broadhurst *et al* [29] to be determinants of specificity of inter subunit interactions. Exploitation of this crucial information in our study probably helps in improvement of prediction accuracy. Identification of specific interacting residue pairs also make the predictions easily amenable to experimental testing by site directed mutagenesis approach. Recent experimental studies [30],[31] have further established the feasibility of altering specificity of inter subunit interactions based on manipulation of putative interacting residues in the docking domain frame work. Apart from helping in deciphering the chemical structure of final polyketide product, our computational analysis of “docking code” in cognate and non-cognate interacting pairs in experimentally characterized modular PKS cluster can also provide knowledge base for fruitfully combining non-cognate ORF pairs for generation of novel aglycone structures. Our analysis of such interacting residues in docking domains of a mycobacterial PKS protein involved in biosynthesis of mycoketide has led to the discovery of a completely novel “Modularly iterative” mechanism of polyketide biosynthesis [48]. However, we must clarify that, apart from interactions between N-terminal and C-terminal docking domains of PKS proteins, the substrate specificity of various catalytic domains would also have a role in preventing chain elongation in case of non-cognate associations of PKS ORFs. Similarly, interactions between ACP and downstream KS will also discriminate non-cognate associations. In this work, we have only addressed the role of docking domains.

## Discussion

We have demonstrated that, the KS domains can be successfully classified into various functional subfamilies with high prediction accuracy using their HMM profiles. Structural modeling of the active site pockets of various iterative KS domains has revealed that certain key residues in the active site pocket can potentially control the size of final product by governing the total number of iterations. This result is in agreement with recent experiments [38],[39] which report cavity volume being a major determinant of substrate specificity of fungal PKSs. The major highlight of our work is that programmed iteration by fungal polyketide synthases may be rationally controlled by site directed mutagenesis of certain specific residues. These results also demonstrate that the number of chain extension reactions catalyzed by an iterative PKS protein can be predicted by computing the cavity volume of the active site pocket of its KS domain. This represents a major advance towards prediction of the polyketide products of iterative PKS proteins.

We have analyzed the docking domain sequences of various modular PKS clusters in detail to investigate if information contained in the docking domain sequences can be used to identify the correct order for channeling of substrates. Using the recently available NMR solution structure [29] of the docking domains from the erythromycin biosynthetic cluster as template, inter polypeptide contacts were analyzed for various types of cognate and non-cognate pairing of ORFs in various modular PKS clusters. Our investigation revealed that, cognate pairing of ORFs always generated energetically favorable inter polypeptide contacts, while in majority of cases non-cognate pairing resulted in energetically unfavorable contacts. The results of our benchmarking on known modular PKS clusters indicated that, using such inter polypeptide contact analysis, it is possible to narrow down the number of possible choices for the cognate order of substrate channeling. Thus our analysis of docking domain sequences would help in predicting the final polyketide products of modular PKS clusters.

In summary, the current work demonstrates that, *in silico* analysis of experimentally characterized PKS clusters can not only enhance our understanding of mechanistic polyketide biosynthesis, it helps in formulating rules for predicting, whether a given PKS protein is modular or iterative, the order of substrate channeling for modular PKSs, and the number of chain extension reactions catalyzed by iterative PKSs. Hence, our results can aid in identifying metabolic products of uncharacterized PKS clusters found in newly sequenced genomes.

## Methods

### KS dataset

In addition to the PKS gene clusters cataloged in the NRPS-PKS server, additional modular PKS clusters that were used for this analysis are ansamitocin [49], albicidin [50], *Bacillus subtilis* PKS, coronafacic acid, compactin CDKS [51], lovastatin LDKS [52], geldanamycin [53], leinamycin [54], lankacidin [55], microcytin (from two organisms) [56],[57], monensin [58], nanchangmycin [41], pederin [59], mupirocin [60], ta1 [61], bleomycin [62] and yersiniabactin [63]. The experimentally characterized fungal type I iterative PKS clusters used in this analysis are aflatoxin [64], avilamycin [65], bikaverin [35], C-1027 [66], calicheamicin (has two type I PKSs) [67], compactin [51], lovastatin [52], fumonisin [68], MSAS from four organisms [69]–[71], sterigmatocystin [72], THN from five organisms [73]–[76], T-toxin [77] and napthopyrone [78]. To this data, we added sequences analyzed in a previous phylogenetic analysis of fungal [79] type-I PKSs.

### KS subfamilies

Profile HMM analysis [33] was carried out by HMMER package. The available KS dataset was divided into five different subfamilies. Apart from the major clusters of iterative and modular KS domains, the KS domain phylogenetic dendrogram showed further clustering into subfamilies like enediynes and non-enediynes within the iterative cluster. Similarly, modular KS domains have three clusters corresponding to pure modular PKSs, hybrid NRPS-PKSs and trans-AT systems. The enediyne family of antibiotics is structurally characterized by the enediyne core, a unit consisting of two acetylenic groups conjugated to a double bond or incipient double bond within the nine-membered or ten-membered ring. The enediyne cores bear no structural resemblance to any characterized polyketides, but precursor labeling experiments have unambiguously established that they are derived minimally from eight head-to-tail acetate units [80]. Natural products of hybrid peptide-polyketide origin have been known for a long time. These are metabolites that are assembled from amino acid and carboxylic acid precursors by hybrid NRPS-PKS gene clusters in which an NRPS-bound growing peptidyl intermediate is further elongated by a PKS module or vice versa [81]. Trans-AT clusters are also referred to as the AT-less clusters. These are complex PKSs where a single AT protein functions in trans- and charges the ACP domains of all the modules in the cluster [20]. Since the modular PKSs often have several KS domains on the same ORF, for building Hidden Markov Models of various subfamilies repartitioning of the various data sets into training and test set was done based on individual ORFs, rather than polyketide clusters or KS domains.

### Modeling of iterative KS domains and analysis of their active site pockets

The various iterative KS domains were modeled using comparative modeling approach. The structural templates were identified by BLAST search against PDB or by using threading approach. Threading analyses were done using a local version of Threader package [82] (downloaded from the PSIPRED protein prediction server site) to identify the structural templates for modeling various KS domains. The various KS domains have been modeled using fatty acid KAS structure as template, which show only about 20% sequence identity with polyketide KS domains. However, availability of several structures of thiolase fold indicates that even at this low sequence identity, two KS proteins can adopt very similar structures. Since the overall active site architecture is conserved in this class of enzymes, our structural predictions are likely to be reliable even at low sequence identity between target and template. The crystal structure of the *act* KS-CLF protein and recently reported structure of DEBS KS have revealed that modular as well as iterative polyketide KS domains also adopt a thiolase fold, thus validating our assumptions.

Models of various polyketide KS domains were built using a local version of modeller V6.2 [83]. Structural mapping, ligand construction and pocket architecture visualization were done using different modules of InsightII package. The active site pockets of iterative KS domains were compared in terms of their hydrophobicity and cavity volumes to understand how binding pocket residues control chemical structure of the polyketide product. Cavity volumes were calculated using CASTp [84]. Only those cavities which contained the catalytic triad residues were chosen from the CASTp output for comparison across various models of a given KS domain. The cavity lining residues (CLRs) were identified from the selected CASTp pockets. The total number and total hydrophobicity of hydrophobic CLRs was tabulated for comparison with the FAS structural template. Hydrophobicity was calculated using Kyte and Doolittle's protein hydropathy scale [85]. Since cavity identification is often sensitive to small changes in orientation of residues, all the above mentioned parameters were calculated from at least five different homology models for the same sequence. Structural alignment of various KS structures was done using Combinatorial Extension (CE) server [86]. Visualization was also done using VMD [87].

### Analysis of docking domains

Secondary structure propensities of various docking domain sequences were derived from the PredictProtein server [88]. ESPript service [89] from the predict protein server was used for structure based sequence alignment of docking domains. Interacting residues for each docking domain pair was identified by aligning their sequences with the docking domain structure. For each interface, the interacting residue pairs obtained from this alignment were ranked as favorable, unfavorable or neutral as per a simple scoring scheme (Table S1). A given combinatorial arrangement of a set of ORFs in a PKS cluster was assigned a score based on the favorable, unfavorable or neutral contacts present in all the interfaces. All the combinatorial possibilities were scored for each modular PKS cluster and score of the cognate combination was compared with scores of various non-cognate arrangements. The computational tool for carrying out inter subunit contact analysis involving docking domains and predicting the order of substrate channeling in modular PKS clusters is available as web server at http://www.nii.res.in/pred_pks_orf_order.html.

## Supporting Information

Figure S1 Dendrogram of active site residues from all KS domains
(0.13 MB DOC)

Figure S2 Superposition of backbones of iterative KS domain models on structural templates
(0.24 MB DOC)

Figure S3 Genomic order vs biosynthetic order
(0.09 MB DOC)

Figure S4 The four helix bundle structure of DEBS docking domain
(0.21 MB DOC)

Table S1 Scoring scheme for docking domain interactions
(0.07 MB DOC)
