# Supplementary material for: Towards Prediction of Metabolic Products of Polyketide Synthases: An In Silico Analysis
Source: PLoS Comput Biol. 2009 Apr 10;5(4):e1000351. doi: 10.1371/journal.pcbi.1000351 (PMC2661021; doi:10.1371/journal.pcbi.1000351)

**Figure S1:**

**Supplementary Figure**:

Dendrogram obtained from a multiple alignment of 47 active site residues extracted from all KS domains. Dendrogram obtained from entire sequence of KS domains also show very similar clustering. Sequences belonging to the five sub families have been depicted in separate colors. Most hybrid (red) and trans-AT systems (blue) have a modular organization and therefore KS domains from these complexes appear to make sub-clusters within the large (green) modular cluster. The enediyne PKSs lie on a distinct branch (orange). KS domains from type-I Iterative PKSs have been depicted in purple color.


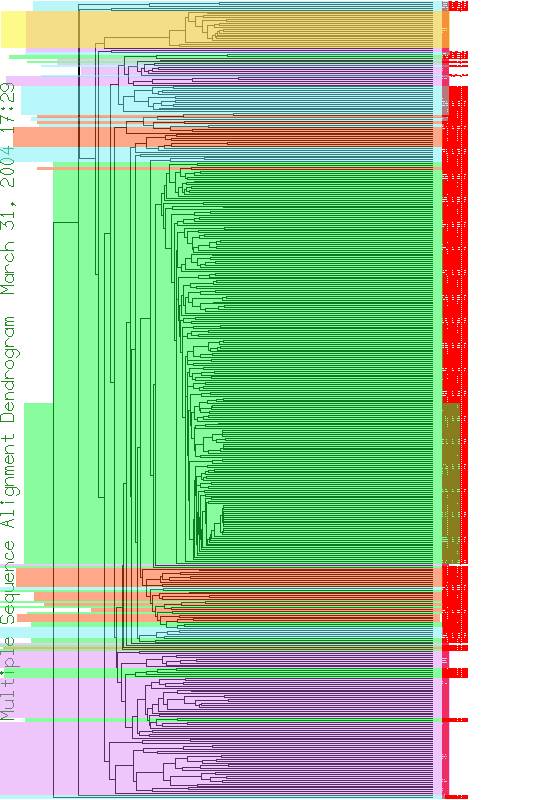

Supplement: Figure S1 — Dendrogram of active site residues from all KS domains (0.13 MB DOC) [file pcbi.1000351.s001.doc]
