# Supplementary material for: Towards Prediction of Metabolic Products of Polyketide Synthases: An In Silico Analysis
Source: PLoS Comput Biol. 2009 Apr 10;5(4):e1000351. doi: 10.1371/journal.pcbi.1000351 (PMC2661021; doi:10.1371/journal.pcbi.1000351)

**Figure S2:**

**Supplementary Figure:** Superposition of backbones of iterative KS domain models on structural templates


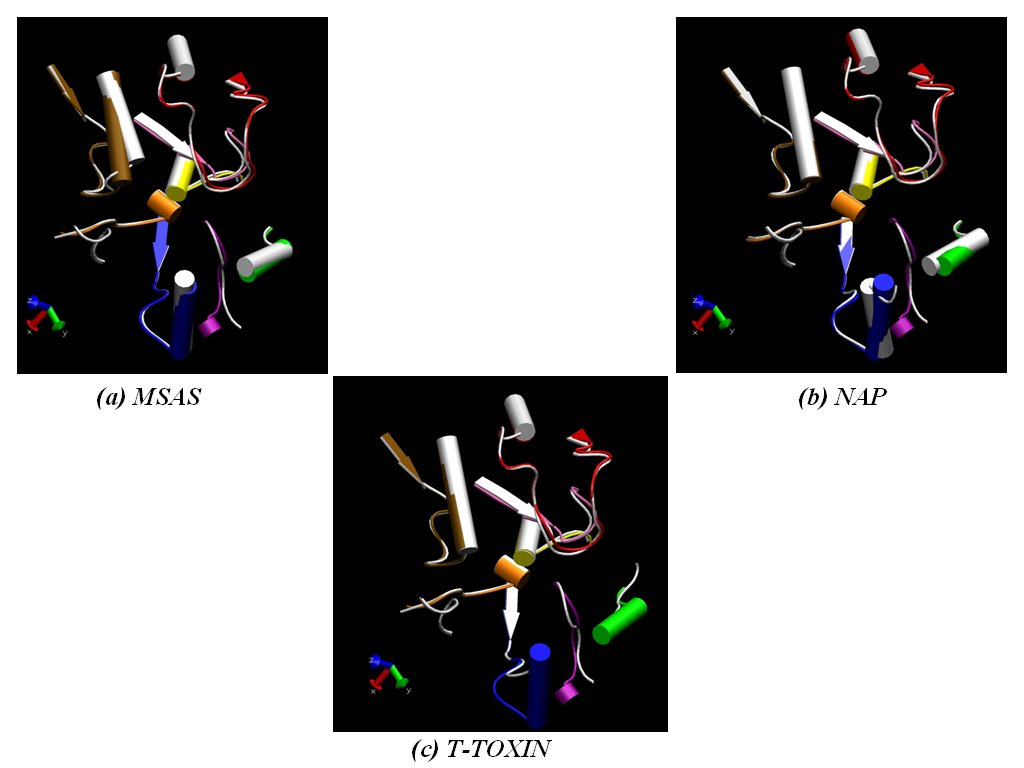

Supplement: Figure S2 — Superposition of backbones of iterative KS domain models on structural templates (0.24 MB DOC) [file pcbi.1000351.s002.doc]
