# Supplementary material for: Towards Prediction of Metabolic Products of Polyketide Synthases: An In Silico Analysis
Source: PLoS Comput Biol. 2009 Apr 10;5(4):e1000351. doi: 10.1371/journal.pcbi.1000351 (PMC2661021; doi:10.1371/journal.pcbi.1000351)

**Figure S3:**

**Supplementary Figure**: This Figure depicts how the order of PKS ORFs in a biosynthetic cluster on the genome can often differ from the actual order in which substrates are channeled by the polyketide synthase. In this gene cluster, the product of modules 1-4 present on the ORF4 is channeled to modules 5-6 present on ORF1.


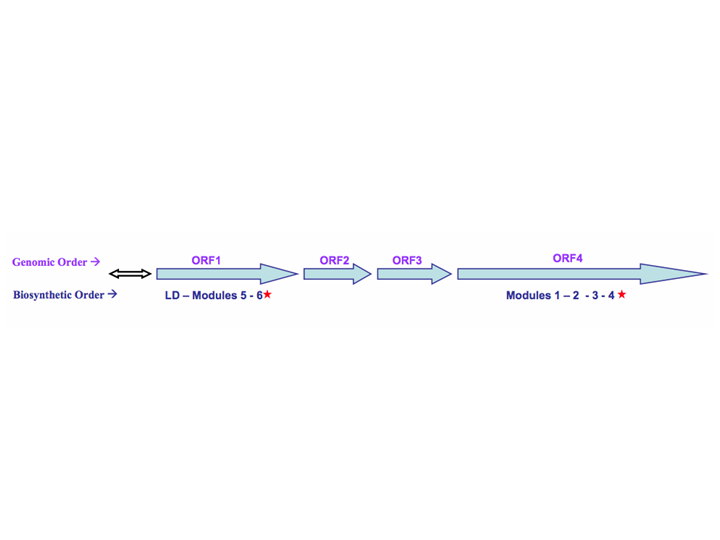

Supplement: Figure S3 — Genomic order vs biosynthetic order (0.09 MB DOC) [file pcbi.1000351.s003.doc]
