# Supplementary material for: Towards Prediction of Metabolic Products of Polyketide Synthases: An In Silico Analysis
Source: PLoS Comput Biol. 2009 Apr 10;5(4):e1000351. doi: 10.1371/journal.pcbi.1000351 (PMC2661021; doi:10.1371/journal.pcbi.1000351)

**Figure S4:**

**Supplementary Figure:**

The four helix bundle structure of DEBS docking domain. The residue pairs giving rise to favorable electrostatic interactions are shown in red and blue space filling spheres.


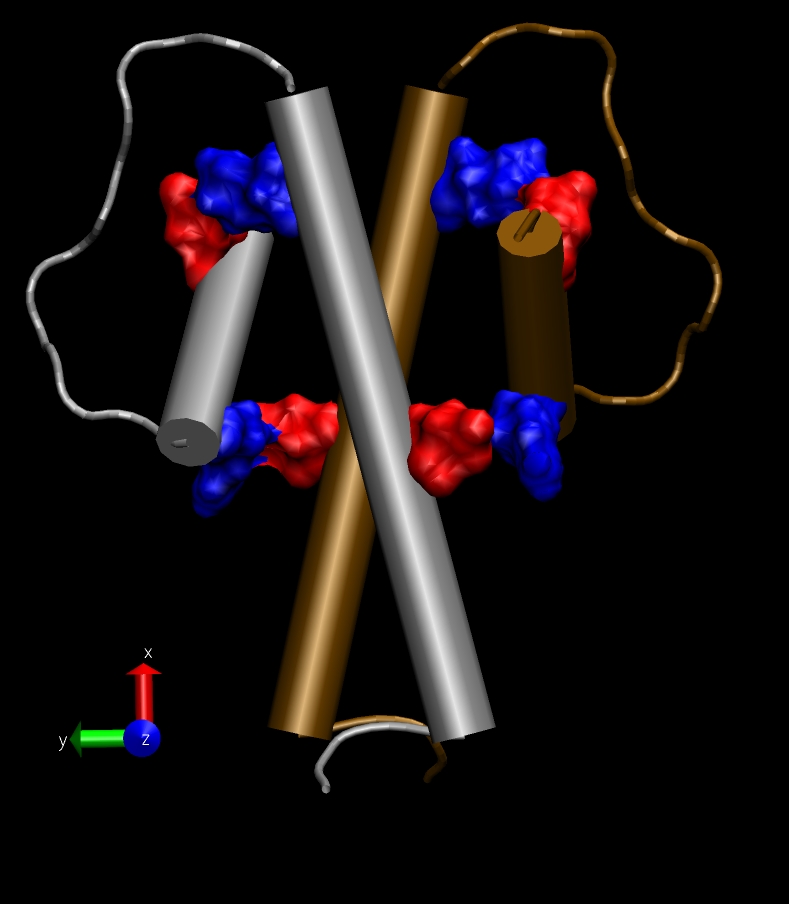

Supplement: Figure S4 — The four helix bundle structure of DEBS docking domain (0.21 MB DOC) [file pcbi.1000351.s004.doc]
