# Supplementary material for: Towards Prediction of Metabolic Products of Polyketide Synthases: An In Silico Analysis
Source: PLoS Comput Biol. 2009 Apr 10;5(4):e1000351. doi: 10.1371/journal.pcbi.1000351 (PMC2661021; doi:10.1371/journal.pcbi.1000351)
Supplement: Table S1 — Scoring scheme for docking domain interactions (0.07 MB DOC) [file pcbi.1000351.s005.doc]

**Table S1:**

**Supplementary Table:**

Scoring scheme for docking domain interactions.

| **Type of Interaction** | **Amino acid Pair** |
| --- | --- |
| Favourable Interactions | Positively charged – Negatively charged |
|  | Negatively charged – Positively charged |
|  | Hydrophobic – Hydrophobic |
| Unfavourable Interactions | Positively charged – Positively charged |
|  | Negatively charged – Negatively charged |
| Neutral Interactions | Any other pair |
